# Interpreting Developmental Surface Dyslexia within a Comorbidity Perspective

**DOI:** 10.3390/brainsci11121568

**Published:** 2021-11-27

**Authors:** Pierluigi Zoccolotti, Maria De Luca, Chiara Valeria Marinelli

**Affiliations:** 1 Department of Psychology, Sapienza University of Rome, 00185 Rome, Italy; 2 Developmental Dyslexia Lab, IRCCS Fondazione Santa Lucia, 00179 Rome, Italy; m.deluca@hsantalucia.it; 3 Department of Clinical and Experimental Medicine, University of Foggia, 71122 Foggia, Italy; chiaravaleria.marinelli@unifg.it

**Keywords:** dyslexia, co-morbidity, reading, acquisition of instances

## Abstract

Recent evidence underlines the importance of seeing learning disorders in terms of their partial association (comorbidity). The present concept paper presents a model of reading that aims to account for performance on a naturalistic reading task within a comorbidity perspective. The model capitalizes on the distinction between three independent levels of analysis: competence, performance, and acquisition: Competence denotes the ability to master orthographic–phonological binding skills; *performance* refers to the ability to read following specific task requirements, such as scanning the text from left to right. Both competence and performance are acquired through practice. Practice is also essential for the consolidation of item-specific memory traces (or instances), a process which favors automatic processing. It is proposed that this perspective might help in understanding surface dyslexia, a reading profile that has provoked a prolonged debate among advocates of traditional models of reading. The proposed reading model proposes that surface dyslexia is due to a defective ability to consolidate specific traces or instances. In this vein, it is a “real” deficit, in the sense that it is not due to an artifact (such as limited exposure to print); however, as it is a cross-domain defect extending to other learning behaviors, such as spelling and math, it does not represent a difficulty specific to reading. Recent evidence providing initial support for this hypothesis is provided. Overall, it is proposed that viewing reading in a comorbidity perspective might help better understand surface dyslexia and might encourage research on the association between surface dyslexia and other learning disorders.

## 1. Introduction

Deficits in reading, spelling, and math are closely associated with each other and with other developmental deficits. This is a phenomenon known as comorbidity. In his seminal paper, Pennington [1] emphasized that we should look at these disturbances in terms of multi- not single-factorial influences. This approach is considerably different from traditional modeling in which different learning deficits were typically examined separately. In fact, specific architectures were proposed for reading (e.g., DRC [2]; the triangle model [3,4]; the CDP+ model [5]) or for math (e.g., the triple code [6,7]). Although these models have different perspectives and are based on different procedural approaches, they all focus on single behaviors and, therefore, provide no clear basis for examining the cognitive factors that are specific to a given behavior and those that might account for the overlap among learning (and other developmental) disorders.

Stemming from Pennington’s [1] proposal, much research has been carried out to try to isolate the cognitive factors that account for the overlap among learning disorders such as dyslexia, on one side, and dyscalculia, different forms of language impairments or ADHD, on the other (e.g., [8,9,10,11]). In these studies, researchers have looked at general cognitive factors, such phonology or cognitive speed, as possible candidates to explain the comorbidity among developmental disorders. While this approach seems promising and has provided some interesting findings, usually no specific attempt has been made in these studies to organize cognitive factors into architectures that are able to predict both specific disturbances and overlap among developmental disorders.

In a recent study we set out to develop a unitary architecture of reading, spelling, and math abilities based on the results of a study which examined cognitive predictors of these behaviors in fifth grade typically developing children [12,13]. In particular, we noted that the breadth of the prediction varied appreciably among different cognitive factors. Some predicted specific behaviors (e.g., either reading, spelling, or math). Others predicted different behaviors but limited to some specific parameters, such as speed. For example, the well-known Rapid Automatized Naming tasks (RAN [14]) predicted reading speed, as well as calculation speed, but not reading or math accuracy. Finally, some cognitive predictors had a widespread influence on behaviors. For example, the ability to do times tables is a powerful predictor of the ability to solve complex calculations; however, it also effectively predicts reading and spelling. Conversely, tests of reading (and spelling) that require the retrieval of memory-specific information (such as performing an orthographic decision task or spelling an ambiguous word) not only predicted reading (and spelling) but also predicted calculation (in particular, accounting for the same portion of variance as the Tables test).

To interpret this complex pattern, we proposed that the cognitive factors which account for learning skills (and potentially for learning disturbances) should be viewed at three separate levels of analysis, i.e., competence, performance, and acquisition. The distinction between competence and performance was originally proposed by Chomsky [15] to account for individual variability of language skills. Chomsky [15] proposed that actual linguistic behavior depends on both “competence,” i.e., an abstract capacity to understand and produce language, and “performance” factors. The latter refer to the fact that the actual linguistic capacity in real-life conditions is task specific, i.e., it depends on factors such as memory, attention, etc. Thus, a key factor that distinguishes competence and performance is that the former is non-task specific while the latter is task specific (Table 1 presents some main characteristics of these levels of analysis related to individual differences in cognitive processing in general and more specifically in reading). Notably, competence and performance cannot be easily distinguished in performance on standard tests and require disentangling ad hoc studies.

We have proposed that a third level of analysis is necessary in order to fully understand actual behavior and we have referred to this as acquisition. Acquisition is the result of learning through practice; practice can influence behavior in different ways. Thus, for a given competence to emerge practice is necessary; this is true even when the competence is to a certain degree genetically controlled, as in the case of language. The first line of the acquisition section in Table 1 refers to this aspect, i.e., to the need for sufficient exposure/practice in order to acquire a competence in a given behavior (such as reading or math). Practice is also essential in the shaping of “performance” factors. The second line in the acquisition section (Table 1) indicates that sufficient practice is required to master the typical task formats that are characteristic of a given behavior. For example, years of practice are necessary in order to read fluently in a left-to-right manner or to write words with smooth, continuous, and fluent hand movements. Some of the performance factors involved in reading are exemplified in the “Functions in reading” column of Table 1. However, practice is also important to acquire item-specific memory traces (or instances) that can be directly used to produce a response (third line in the acquisition section in Table 1). Indeed, in many situations previously acquired information speeds up the solution of a given task, i.e., it allows automatizing performance. For example, in doing complex computations, we do not always use arithmetic algorithms but typically bypass them by referring to known “arithmetic facts” (such as tables) to make calculations smoother and faster. Importantly, it can be observed that this capacity of the cognitive system to make automatic responses through reference to item-specific memory traces does not fit with the learning of abstract rules (which builds up a competence in a given cognitive domain) and the learning of task-specific requirements (which improve performance in daily activities), indicating the need to examine acquisition level separately [13].

The ability to use previously acquired inform ation to speed up processing in complex tasks has been the subject of much research. For example, in a series of investigations, Logan [16,17] proposed that the ability to automatize is developed through the acquisition of specific instances through practice. This researcher developed the “Instance theory of automaticity”. The instance theory makes three fundamental assumptions concerning the processing of an object or event: 1. obligatory encoding; 2. obligatory retrieval; and 3. instance representation. Following Logan [17],

“When people perform the same task repeatedly, obligatory encoding causes instance representations of the same act to be stored in memory. The more repetitions, the more instances are stored. Obligatory retrieval causes information to become available when familiar situations are encountered once again. The more instances there are in memory, the more will be retrieved (i.e., the response from memory will be stronger). The assumption of instance representation allows one to model the retrieval process as a race in which the fastest trace determines performance (i.e., performance is based on the first instance to be retrieved from memory).”

Notably, according to this theory, response times become faster according to a power function, a point developed in greater detail below. Our working hypothesis has been that tasks calling on the retrieval of specific instances may play a key role in accounting for the comorbidity of learning disorders.

## 2. Aims of the Present Report

Within this perspective, we propose a model that could account for the partial association/dissociation among learning behaviors and that could serve as a heuristic for interpreting the comorbidity of learning disorders [13]. In this concept paper, we wish to present the reading part of this model in greater depth and to illustrate how it accounts for reading processes differently from other current models of reading. Secondly, we will attempt to show that this model might lead to a fresh view of the long-lasting controversy over the interpretation of surface dyslexia, i.e., a reading disorder in which the individual is unable to read words as a whole and retrieves their pronunciation by applying phoneme-to-grapheme conversion rules. In particular, as described in greater detail below, we propose that, when reading is viewed within a comorbidity perspective, it is possible to see surface symptoms as due to a general domain factor that accounts for the partial association among reading, spelling, and math.

### Modeling Reading within a Co-Morbidity Perspective

**Figure 1:**
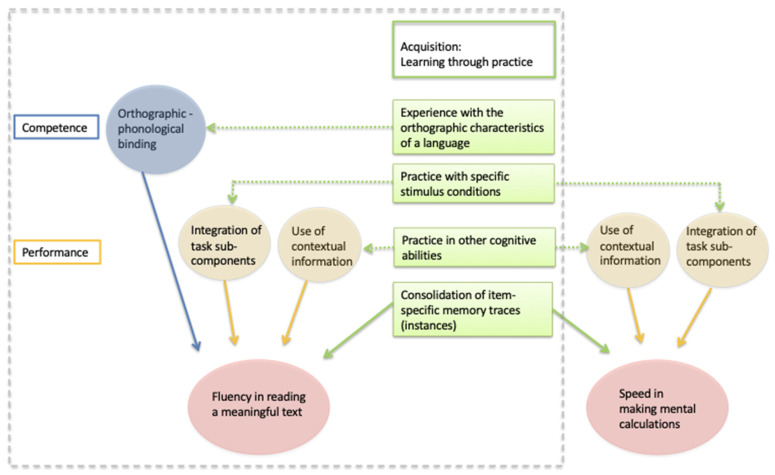
A multi-level model of the fluency in reading a meaningful text (see area within the grey dotted rectangle). The model envisages the separate influence of competence, performance, and acquisition. The figure especially focuses on the analysis of the acquisition level, i.e., the different ways of learning by practice (illustrated by four green boxes). The acquisition of reading competence is hypothesized in terms of orthographic–phonological binding derived from prolonged experience with orthographic material (top box). Two boxes report two examples of how practice may influence the performance level in reading (integration of task sub-components and use of contextual information). The bottom box reports the influence on reading behavior directly through the recall of item-specific memory traces (instances), favoring reading automatization. The right portion of the figure shows some of the connections of the acquisition boxes with a behavior different from reading (speed in making mental calculation). Note that some performance factors may exert similar influences on more than one behavior (although there may also be performance factors with different influences on different behaviors; see main text for further discussion of this point). Furthermore, it is expected that the recall of item-specific memory traces may directly boost performance across different behaviors. Finally, no connection is envisaged between the reading competence and the performance in making mental calculations.

Figure 1 presents a multi-level model of reading. The model aims to account for reading performance in a naturalistic task, such as fluency when reading a meaningful text aloud. This perspective is different from traditional modeling, which is focused on describing the abstract competence of reading and typically neglects to specify how such competence is expressed in naturalistic tasks (for a discussion of this point see [18]). Fluency in reading a meaningful text is influenced by a variety of predictors (details can be found in a previous study [12,13]), which act at different levels (competence, performance, and acquisition).

It is hypothesized that the reading competence is linked to a process of orthographic–phonological binding. Both our empirical findings [12,13] and theoretical considerations underlie this choice. In particular, a large literature indicates that the binding of orthographic and phonological processing may be the key competence for reading (summarized in [19]). Support along these lines comes also from lesional and imaging data which show that, through appropriate stimulation [20], an area in the left occipito-temporal sulcus becomes attuned to the orthographic regularities of a given language. Critically, this so-called Visual Word Form Area (VWFA) is not responsive to specific words but rather to frequent letter combinations [21], thus providing a pre-lexical neural substrate for forming a “graphemic description” (see also [22]).

It can be hypothesized that several factors (including executive functions, attention, progress monitoring, or motivation) contribute to the performance in a naturalistic task. For the sake of presentation only two performance factors are exemplified in Figure 1: the integration of task sub-components and the use of contextual information. As to the former, to read effectively a child has to learn to synchronize a number of ongoing processes. These include the ability to process the fixated visual image, anticipate the size of the next (usually rightward) saccadic eye movement, and hold in memory the recognized word to be uttered. This complex pattern of processes needs to be effectively managed by children so that they can achieve the reading fluency adequate for their age and experience. The RAN [14] task captures this complex skill well because it encapsulates the whole reading task except for orthographic analysis [23]. Children with dyslexia show deficits on RAN tasks, which are difficult to interpret in terms of reading competence (for a review see [24]). Note that “integration of task sub-components” is in itself a macro factor that recapitulates a number of partially independent processes. Thus, a full model of reading would require also taking into account how text decoding (and uttering) is integrated in the programming and execution of eye movements. Models such as the EZ Reader [25] or the OB1-reader [26] are formal attempts to model eye movement programming along with reading decoding processes. Still, for the specific purpose of accounting for developmental reading deficits, this macro-factor captures individual variability well as evidence indicates that children with dyslexia are spared in programming and executing eye movements as such [27,28,29,30,31]. Experience in reading also expands the child’s ability to use contextual information to facilitate the processing of ongoing stimuli in situations such as reading a text. The context has a top-down influence on processing and its influence has been shown in both typically developing children and children with dyslexia [32].

Finally, it has been proposed that performance on a naturalistic reading task also depends on the possibility of directly accessing item-specific memory traces (or instances) obtained through prolonged practice. It has been proposed that “remembering particular events and their details through mnemonic specificity” is a key and general function in learning [33]. Accessing specific memory traces acts by boosting performance favoring automatic responding.

Overall, the model predicts that performance on a functional task, such as reading a text, depends on the joint influence of different factors acting at different levels of processing. Note that the model in Figure 1 is only partially developed. As stated above it does not explicitly consider the role of eye movements or executive function; moreover, it does not focus on visual processing, which is preliminary to any form of acquisition subtending reading; finally, it is specific for the task/measure considered (i.e., fluency in reading a text). These features may reduce its generality. However, the critical point here is that the model helps to put together factors which influence reading at different levels of processing, some of which are typically left out in the rarefied search for the reading code within traditional models of reading. 

Furthermore, the model permits seeing reading along with other learning behaviors. In Figure 1, this is exemplified by showing some relationships that contribute to accounting for the overlap in reading and math skills and potentially could explain the comorbidity of dyslexia and dyscalculia (for a full model of reading, spelling, and math see [13]). Thus, recall of item-specific memory traces (instances) is expected to influence speed in making mental calculations as it favors reading fluency; thus, it is an important factor in the comorbidity of learning disorders. However, overlapping is also expected at the performance level; for example, the integration of task sub-components also has a role in the skill of making mental calculations. Consistently, a large review has shown that RAN tasks predict fluency (more than accuracy) in making calculations [34]. Similar considerations may apply to the ability to use contextual information to favor mental calculations [13]. However, other “performance” factors may act differently on different behaviors, e.g., it has been reported that spatial short-term memory is critical for distinguishing between children with and without numerical deficits, after controlling for several factors, including age and IQ [35]; however, it is not generally considered critical in the case of reading. Notably, reading competence is not expected to predict the ability of making mental calculations. Thus, co-morbidity among learning disorders can be best appreciated by breaking down relationships at different levels of processing.

Finally, one important feature of the model is that it also allows framing the role of practice. In a recent review that was partially based on an analysis of the reading of illiterates and children with dyslexia, Huettig et al. [36] emphasized that in the attempt to understand developmental dyslexia, it is extremely difficult to discern specific causes from reduced and/or suboptimal reading experience. The proposed model specifies that practice acts in different ways at different levels. It has been proposed that separating these different practice effects could be critical for establishing the different (but possibly concomitant) causes of dyslexia.

## 3. Interpretations of Surface Dyslexia in Acquired and Developmental Cases

One area of prolonged debate among models of reading concerns surface dyslexia. In surface dyslexia (whether acquired or developmental [37]), the individual has difficulties in reading the word as whole (the whole word) and tends to process it through the application of phoneme-to-grapheme conversion rules. Thus, surface dyslexics typically present regularization errors in the case of irregular words (such as bear → beer). It is interesting to reflect on the interpretations of this reading profile that derive from other models of reading and the present proposed model.

In order to account for the specific incapacity to respond to items that cannot be solved through grapheme-to-phoneme conversion (such as irregular words), the DRC model [2] proposes the presence of two partially independent routes, one of which is concerned with the activation of an orthographic lexicon. A selective deficit in reading irregular words indicates that the lexical route is damaged and is diagnostic of surface dyslexia [37,38,39]. This interpretation of surface dyslexia has constituted a key challenge between theorists in the acquired [40] as well as in the developmental forms of the disturbance [41,42]. Differently, researchers working within the triangle model [3,4] have generally emphasized the continuity between reading pseudo-words as well as regular, regular-inconsistent (words, like “hoot”, which take the most frequent pronunciation of their body neighborhood, although other words with the same body have a different, although more infrequent, pronunciation), and irregular words, pointing to a single reading mechanism dealing with regular, regular-inconsistent words, and irregular words (see [40]). According to this view, the deficit in reading irregular words is not a selective, isolated disorder.

Within the triangle model perspective, acquired surface dyslexia is also explained in terms of some degree of inefficiency in the interaction between phonological and semantic representations [40]. Although there is some evidence in favor of this hypothesis [43]; cases of dissociation between semantic dementia and surface dyslexia have also been reported ([44]; for a discussion of this question see [45]). Note also that the role of semantic processing in reading aloud has been questioned in general [46] and more specifically in the case of regular orthographies, such as Italian [47,48].

In a developmental perspective, some researchers have proposed that surface dyslexia might not be a selective deficit but merely a reading “delay” ([49]; for a reply see [50]). Data derived from the so-called reading-match control paradigm seem to point to this conclusion (e.g., [41,42]). Thus, cases of children with surface dyslexia diminish dramatically if they are compared to children matched for their reading level (rather than chronologically). Based on these observations, some authors have proposed that surface dyslexia is due to nothing more than “lack of exposure to print” (e.g., [42]).

However, there are several reasons to question this conclusion. First, it is well known that children with dyslexia do not like to read (e.g., [51]). However, this is a general tendency, and it is not clear why in the quoted studies (e.g., [42]) some children show a pattern of surface dyslexia as an effect of this and others a pattern of phonological dyslexia. In fact, even though several approaches have been developed to examine reading habits and their strength in predicting reading efficiency has been repeatedly tested (e.g., [52]), very few studies have directly examined whether exposure to print is specifically linked to surface dyslexia. Possibly the most informative investigation in this respect is that of Peterson, Pennington, and Olson [53]. These authors compared large groups of children with surface or phonological dyslexia using a widely adopted measure of exposure to print (i.e., the Author Recognition test). The latter requires the recognition of the names of real books among a number of foils, i.e., invented titles) developed by Stanovich et al. (e.g., [54]). Notably, there was no difference between the amount of print exposure of children with surface or phonological dyslexia (Study 2 [53]). Second, the reading-match design has been severely criticized as inherently flawed [55,56]. In particular, it has been shown that it is based on the unproven (and unlikely) assumption that improvement in reading different classes of stimuli (such as words and pseudo-words) with increasing experience is homogeneous (for a discussion see also [57]). As an effect of this, it is biased toward finding putatively “specific” deficits for stimuli/conditions associated with slower rates of learning (as is the case for pseudo-word items) and/or higher levels of inter-individual variability (again as in the case of pseudo-word items). By contrast, it has been shown that if unbiased control groups are used, a large number of children show a surface-like performance pattern [58].

Overall, the interpretation of a surface-like profile has been a key issue in choosing among alternative interpretations of dyslexia. On one hand, in the DRC tradition surface dyslexia is a selective deficit in the acquisition/activation of the lexical route. Thus, the lexical route is intrinsically part of reading competence, which depends on the foundation of two partially independent mechanisms. On the other hand, the connectionist tradition aims to simplify the conception of reading competence by assuming a unitary mechanism. In this perspective, surface dyslexia is somewhat difficult to interpret and there is a tendency to dismiss this reading profile as non-specific, e.g., seeing it as a mere delay in acquisition possibly due to reduced reading experience.

The reading model illustrated in Figure 1 approaches this problem in a way that is distinct from both approaches. On one hand, it accepts the idea that a surface-like pattern of reading is a “real” deficit, in the sense that it is not due to an artifact, such as limited exposure to print. On the other hand, it proposes that the ability to consolidate specific traces (or instances) is a domain-general mechanism that the cognitive system uses to automatize performance, not a mechanism specifically linked to reading competence. Thus, in this view, surface dyslexia is just a component of more general difficulty in acquiring (or retrieving) specific memory traces, not a deficit confined to reading.

It must be added that this is still a working hypothesis that still requires supporting evidence, although some already available results seem to fit well with it. One clear prediction is that children with surface dyslexia should also have difficulty in retrieving specific memory traces in spelling and math. Some evidence fits well with this idea. For example, it has been reported that adults with dyslexia were impaired in retrieving arithmetic facts although their numerical representations were spared [59]. We would expect individuals in this condition to show a predominant pattern of surface errors in reading; however, this prediction was not directly tested in the original study [59]. In a study on the association between reading and spelling, it was reported that children with dyslexia showed parallel, item-specific deficits in spelling words with an ambiguous transcription and in making orthographic judgments about the very same items [60]. In a replication and extension of these findings, it was reported that in spite of their item-based lexical deficit children with dyslexia showed spared sensitivity to the distributional information of sound-spelling mappings [61]. Overall, these selective associations are in keeping with the idea that defective ability in consolidating individual instances could impair reading performance, as well as that of spelling and math, even in the absence of a deficit of competence as such.

We recently obtained further evidence along these lines in a study in which we examined performance on standard clinical instruments in a sample of typically developing children and used the network analysis to obtain a unitary picture of reading, spelling, and math skills [62]. The network analysis indicated that the different measures of the same ability (i.e., reading, spelling, and math) formed separate clusters, in keeping with the idea that they are at least in part related to separate competences. However, results also indicated that tests requiring the ability to recall specific instances (such as the Arithmetic facts subtest and Spelling words with ambiguous transcription) showed nodes with higher strength centrality in the connection among these clusters, a finding in keeping with the idea that the ability to consolidate specific instances might be crucial for understanding the overlap among learning skills (and potentially also disturbances).

### Outcome versus Process Measures of the Individual Ability to Consolidate Specific Instances

It seems important to emphasize the complexity associated with examining deficits due to a low ability to consolidate individual instances. Deficits in reading (or spelling) irregular words, as well as retrieving arithmetic facts, represent outcome measures of a long-lasting process of acquisition. Therefore, it would be important to obtain measures of the process of automatization through instance acquisition.

Only a few studies have examined the acquisition of new materials in children with dyslexia and controls. Most of this research is concerned with the task of learning a set of non-words [63,64,65,66]. Typically, control children show a clear sensitivity to the length of the stimulus, which disappears by the end of training, indicating acquisition of individual nonwords. In keeping with the idea that automatization is item-specific [16,17], learning does not directly extend to new sets of nonword stimuli (e.g., [66]). Children with dyslexia show generally slower learning and, by the end of training, they are still sensitive to the effect of length [63,64,65]. Thus, these findings are in keeping with the idea that the ability of children with dyslexia to acquire individual memory traces is impaired.

Still, most studies are restricted to reading and do not provide information as to whether the ability to consolidate individual instances represents a domain-general factor, as predicted by the model. To this aim, we recently completed a study [67] in which we examined the performance of typically developing fifth graders during the learning of an invented rule. In this novel paper-and-pencil task, the child saw a matrix of letters, and for each letter had to write another letter two letters ahead in the alphabet (e.g., see letter A, write C). This task could be solved based on an algorithm or, with prolonged practice with the same target letters, with reference to specific instances. Both group and individual performances indicated a power function rate of learning, as expected [17]. We used the parameters of the individual power function fits to predict performance on tasks requiring knowledge of individual items (such as doing tables or retrieving lexical representations) as well as on tasks requiring the application of rules or algorithms (such as judging numerosity or spelling through sub-lexical mapping). In keeping with the hypotheses, indicators of the individual rate of learning individual instances predicted performance on the former but not the latter, tasks. Critically, these predictions were present independent of domain, i.e., they were present for reading, as well as for spelling and math. Therefore, these results represent a first piece of evidence that the ability to consolidate individual instances represents a domain-general factor that could be particularly important in accounting for the overlap between learning skills (and potentially for the comorbidity of learning disturbances).

## 4. The Ability to Consolidate Specific Traces: A Route to Understand Surface Dyslexia

The interesting point here is that one should not necessarily assume that the child/adolescent has had limited exposure to print. Rather, it seems more interesting to propose that it is the “individual sensitivity to the exposure to print” that is the critical variable in producing a surface-like pattern of reading. In this way, the interpretation moves away from the stimulus side to the individual’s processing capacity. One may reasonably assume that children vary in their ability to optimize their performance to the repeated observation of individual events (in this case, words).

Note that this interpretation does not undermine the importance of the role of practice (or exposure to print) as such [36]. Rather, it emphasizes that children can show marked individual differences in learning instances even in cases (such as the experimental conditions of Logan [17]) in which the amount of practice is kept constant. The fact that children with dyslexia typically do not engage in reading (e.g., [51]) may well amplify their problem in reading. Therefore, in everyday, uncontrolled conditions, it would be more exact to say that the impact of practice over the learning of specific instances (i.e., words) is expected in the interaction between individual sensitivity to exposure to print on one hand and actual amount of exposure on the other.

### Acquisition of Specific Instances

In the perspective of the instance theory of automatization, Logan [16,17] reported that performance moves from the use of an algorithm to the direct identification of specific “instances” following a power function relationship, i.e., a curve characterized an initial sharp improvement and a subsequent progressively decreasing rate of improvement. It is intriguing that Logan [17] also demonstrated that a power function relationship exists for all percentiles of a given distribution, not only for the average group performance. This indicates that children in the lower quartiles of performance might also improve their performance by a power function curve. An interesting point that derives from this observation is that the same shape of this relationship permits establishing the time evolution of the reading difficulties associated with a low ability to consolidate instances (i.e., words). In particular, it is expected that children with lower skills in reading will need much more time to optimize their performance and that the time lag between an impaired performance and that of an average group of children with typical development will actually increase (not decrease) over time.

**Figure 2:**
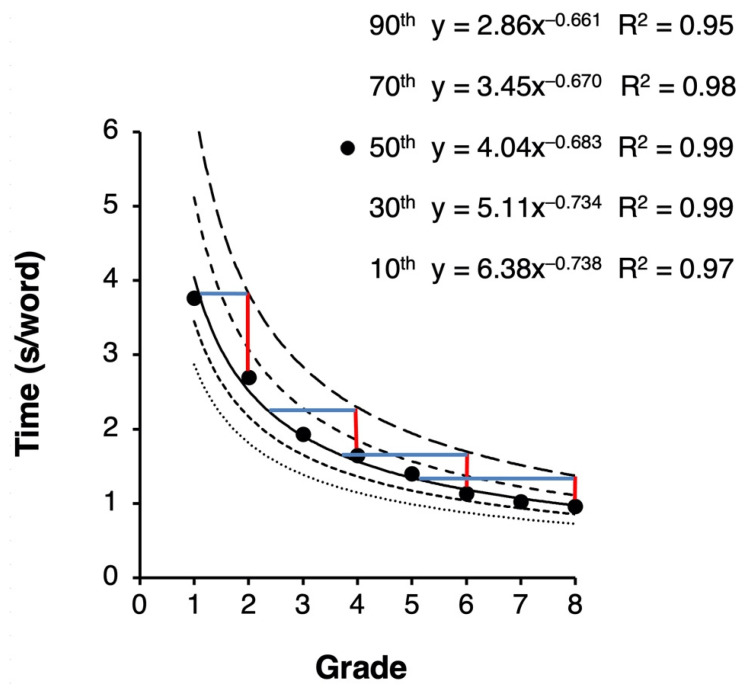
Reading times (in s/word) in the reading of a list of 30 high-frequency words. Data (redrawn from [66]) refer to a cross-sectional study on a group of 1st to 8th grade typically developing children. Power functions indicate performance at the 10th, 30th, 50th, 70th, and 90th percentiles (curves from top to bottom). Vertical red bars mark the difference between the 90th and the 50th percentile for each grade; note that the difference becomes quantitatively smaller at progressively higher grades (as indicated by shorter red bars). Horizontal blue bars link the performance corresponding to the 90th percentile with the corresponding performance at the level of the 50th percentile; this indicates the distance (in years) between the two levels of performance. Note that the distance (in years) increases at progressively higher grades (as indicated by longer blue bars).

An illustration of this relationship with regard to reading is presented in Figure 2 and based on data (redrawn) from a previous cross-sectional study by our group [68]. Data refer to the performance of children in first to eighth grade in reading a list of 30 high-frequency long words (expressed in terms of s/word). In keeping with Logan’s [17] instance theory of automatization, data points for different percentiles (10, 30, 50, 70, and 90) all closely follow a power function relationship (at least R^2^ = 0.95) with similar coefficients. Note how the absolute difference between a low (90th percentile) and an average (50th percentile) performance decreases with increasing grades (i.e., as an effect of practice). This is exemplified in grades 2, 4, 6, and 8 with a red vertical bar (differences were 1.37, 0.56, 0.59, and 0.44, respectively). By contrast, the time lag for the performance at the 90th percentile with respect to that of the 50th percentile actually increases over time. This is exemplified in grades 2, 4, 6, and 8 with a blue horizontal bar. Thus, when compared to performance on the 50th percentile, performance at the 90th percentile in grade 2 indicates a one-year delay; in fourth grade, performance in the 90th percentile indicates an approximately two-year delay; the same comparisons indicate a two-and-half year delay for performance at the 90th percentile in sixth grade and a three-year delay in eighth grade.

This analysis indicates that the shape of the learning curve [16], as well as the fact that it applies to the whole distribution [17], allows making predictions about the timing of the emergence of possible deficits in lexical processing. In particular, one expects difficulties in lexical processing to emerge most clearly late in reading acquisition. Evidence on this topic is still scanty. Some studies have reported evidence about late emerging word recognition problems, but they are not detailed enough to establish whether the dyslexics involved showed a surface profile (e.g., [69]). It can also be observed that single case studies of developmental surface dyslexia usually do not refer to young children. The original case (i.e., CD) was tested when the subject was 15 years old [37]. Out of a total of six cases, Temple’s early series [70] included three developmental dyslexics (Ph.B., 12.9 years; SL, 12 years; and PB, 10.5 years of age). Most subsequent single case studies referred to subjects tested at an older age (e.g., “undergraduate” [71]; Allan: 22 years [72]; Mandy: “early twenties” [73]; DF = 11.4 years [74]; and MI: 9 years of age [39]). Friedmann and Lukov [75] presented an extensive single case series of Hebrew-speaking developmental surface dyslexics; most subjects were between 10.7 and 15.7 years of age (12:1); two adult dyslexics (21 and 43 years of age) were also reported. Clearly, this evidence is only suggestive as it might be associated with various factors affecting the time of testing. Still, it is suggestive that the youngest child with surface dyslexia extensively tested appears to be MI who was examined when 9 years old [39]. It should be added that group studies comparing phonological and surface dyslexics provide no clear information on this point as age is usually pre-determined and homogeneous for the two groups [38,41,42,58]. One study which directly tested the time of emergence of a surface pattern was carried out in Italian, a language which is highly regular in the phoneme-to-grapheme conversion but moderately irregular in the phoneme-to-grapheme direction with a substantial number of words with unpredictable transcription. Angelelli et al. examined children with dyslexia with regard to their capacity to spell regular and irregular words (as well as nonwords) [76]. In third grade, children with dyslexia showed an undifferentiated pattern of spelling impairment that included regular words, regular nonwords, and words with unpredictable spelling. By contrast, fifth grade children showed a prevalent impairment in writing words with unpredictable transcription (with a high proportion of phonologically plausible errors). This indicated the predominance of a lexical deficit late in literacy acquisition. Overall, the available evidence is limited but not inconsistent with the idea that deficits in lexical acquisition can emerge late in reading acquisition. Several studies of English children examined the relative proportion of phonological and surface dyslexia [38,41,42,58]. It might be expected that the relative proportion of surface dyslexics will be higher in older groups of subjects, a prediction still not directly tested.

The above-described characterization of surface dyslexia is based on time measures, whereas key studies of this disturbance focus on accuracy measures, in particular on accuracy in reading irregular words (in English). However, there are also classical studies of children with surface dyslexia which illustrate their performance with reaction time measures. In fact, in a series of investigations Seymour and colleagues (e.g., [77,78]) reported that these children (whom they called children with “morphological” dyslexia) showed a particularly strong sensitivity to length effects in their reading RTs, which we proposed to be a marker of the fractionated, parceled way of reading [79], i.e., reading by an “algorithm” rather than by “sight”.

Overall, we propose that some children may suffer from defective acquisition of instances (i.e., they are slow/inefficient in learning new words). At least in principle, this could occur in the absence of a deficit in the core competence of reading; thus, a child might have some ability to read through orthography-to-phonology conversion but be unable to take advantage of practice, as much as other children do, by directly memorizing words. Eventually, this would produce a pattern of performance characterized by a lack of automaticity, which could be manifested as reading slowness [79]. Furthermore, reduced ability to automatize responses to individual words might be expressed as reduced lexical expansion, i.e., the number of words that generate a direct obligatory activation might be relatively few and limited to those with high or very high frequency [60]. As a result, the word frequency effect might actually be quite strong, indicating the possibility of a deficiency in the direct retrieval of low-frequency words (e.g., [80]). In the particular case of English, difficulty reading words that have to be learned individually is particularly clear in the case of irregular words, as is typically reported in surface dyslexia. Note that this type of practice effect concerns optimization of the response to individual words and should be distinguished from the effect of practice over the tuning of the competence in orthographic–phonological binding (referred to above), which is a general, abstract acquisition of the regularities of a given orthography (not of specific words).

## 5. Conclusions

Here we advanced the working hypothesis that a surface-like pattern of reading is a “real” deficit in the sense that it is not due to an artifact, such as limited exposure to print, and does not represent specific reading difficulty but is a cross-domain defect in taking advantage of practice. Thus, this deficit is not expected to be confined to reading but should extend to other learning behaviors, such as spelling and doing math. We presented recent evidence that provides initial support for this statement. However, much research is needed to test the relationship. In particular, it is possible to predict that individuals who show a surface dyslexia pattern will also show selective deficits in tasks requiring the recall of individual memory traces, such as learning math tables. To our knowledge, this prediction has not yet been systematically tested.

Surface dyslexia is hypothesized to arise from a general-domain low ability to consolidate instances. In the case of reading, this is expressed as inadequate lexical expansion. In irregular orthographies, such as English or Hebrew, this should be expressed most clearly as a deficiency in reading irregular words; in regular orthographies, such as German or Italian, this should be expressed as difficulty in fluently reading low frequency words. Notably, placing surface dyslexia within the instance theory of automatization [16,17] allows for the prediction that a surface dyslexia profile should emerge most clearly at an advanced stage of learning, a prediction that has still not been systematically tested. It may be added that understanding the nature and time development of lexical impairments in surface dyslexia could be instrumental for planning individually tailored intervention programs for the amelioration of the disturbance.

In conclusion, here we propose that viewing reading in a comorbidity perspective might help us to improve our understanding of surface dyslexia and might foster research on the association between surface dyslexia and other learning disorders.

## Figures and Tables

**Table 1:** Main functions and characteristics of competence, performance, and acquisition levels as related to individual differences in cognitive processing in general and more specifically for reading. In the last column, the expectation in terms of dissociation/association for deficits at different levels is spelled out, e.g., it is expected that competence deficits in different domains will dissociate from each other whereas deficits in automatization will be found in different domains (see text for a more thorough presentation). In the table, “domain” is a broad term that indicates areas of cognitive function (such as language or spatial orientation as well as reading and math). “Behavior” refers more restrictively to action, or function, that can be objectively observed or measured in response to controlled stimuli. The term “task” refers to the peculiar characteristics of the goal-oriented activity undertaken by the subject, such as reading aloud a sentence from left to right on a static display. The term “item” refers to the specific instance considered, such as a given word (e.g., “mother”) in the case of reading.

Level of Analysis	General Function(s)	Main Characteristics	Functions in Reading	Expected Dissociation/Association
Competence	Activation of a specific set of representations and processes	-Domain-dependent -Task-independent -Sensitive to practice	Word reading based on orthographic–phonological binding	Dissociation between deficits in different domains
Performance	Optimizing behavioral efficiency in everyday conditions	-Task-dependent -Partially domain-dependent -Sensitive to practice	-Left to right scanning -Programming saccadic eye movements for optimal landing position -Integration of decoding with utterance production -Use of contextual information, etc.	Association or dissociation depending on high/low similarity of task characteristics
Acquisition: learning through practice	Emergence of a competence in a given domain (e.g., language, reading) through exposure and/or explicit teaching	-Domain-dependent	Building reading competence	See competence
	Mastering of typical task formats that are characteristic of a given behavior	-Partially domain-dependent	Tuning reading performance in everyday complex reading tasks	See performance
	Automatization of performance, through the activation of specific memory traces (instances)	-Item specific -Domain-independent	Building a repertoire of words with immediate and obligatory retrieval and recognition (orthographic lexicon)	Association among deficits in different domains, including learning disturbances (comorbidity)

## Data Availability

Data will be made available only upon request for reasons of privacy.
